# Computed Tomography Angiography Recognition of a Hemorrhagic Moyamoya Pattern May Alter Acute Blood Pressure Management

**DOI:** 10.7759/cureus.112610

**Published:** 2026-07-13

**Authors:** Madison N Sheppard, Alexandria Zarilla, Lindsey Mayer, Cristin Sampson

**Affiliations:** 1 Department of Medicine, Lake Erie College of Osteopathic Medicine, Bradenton, USA; 2 Department of Internal Medicine, Lee Memorial Hospital, Fort Myers, USA

**Keywords:** acute hemiplegia, blood pressure management, interventional management, interventional radiology, intracerebral hemorrhage, m1 middle cerebral artery segment, moyamoya disease (mmd), neuroradiology procedure, radiology and interventional radiology

## Abstract

Moyamoya disease (MMD) is a rare progressive cerebrovascular occlusive disorder characterized by stenosis of the terminal internal carotid arteries and the development of fragile lenticulostriate collateral vessels. In adults, MMD can manifest as intracranial hemorrhage (ICH), carrying significant morbidity and a high risk of recurrent bleeding. When hemorrhagic MMD coexists with bilateral large vessel occlusion, acute management becomes exceptionally complex, as the standard imperative to aggressively lower blood pressure following ICH directly conflicts with the hemodynamic requirements of collateral-dependent cerebral perfusion.

A 52-year-old Caucasian woman with hypertension and treated hypothyroidism, recently started on aspirin for TIA-like symptoms, presented with acute-onset left hemiplegia and a National Institutes of Health Stroke Scale (NIHSS) score of 11. Hypertension was her sole conventional atherosclerotic risk factor, and thyroid autoantibodies obtained previously had been negative. Non-contrast computed tomography (CT) identified a 2.3 × 1.9 × 2.5 cm right gangliocapsular intraparenchymal hemorrhage with a concurrent small right thalamic hemorrhage. Computed tomography angiography (CTA) demonstrated bilateral M1 segment occlusions with distal M2 reconstitution, a pattern that immediately raised concern for MMD and led directly to the recognition that mechanical thrombectomy was not indicated. The neurosurgical team concurred with a clinical and radiographic diagnosis of moyamoya vasculopathy on hospital day two. Rather than applying standard post-ICH targets, the team individualized blood pressure management to a permissive systolic range of 120 to 160 mmHg, chosen to balance limiting hematoma expansion against preserving collateral-dependent perfusion. Intravenous nicardipine was successfully weaned by hospital day three. The diagnosis was considered probable and imaging-based. Formal cerebral angiography for Suzuki staging and revascularization planning was appropriately deferred during the acute admission and planned on an outpatient basis; the patient was discharged to an inpatient rehabilitation facility on hospital day five but was subsequently lost to follow-up, so digital subtraction angiography (DSA) was never performed.

Bilateral M1 occlusion with distal M2 reconstitution on CTA in the setting of gangliocapsular hemorrhage should raise suspicion for MMD and may inform both interventional and hemodynamic decision-making. Recognition of the characteristic CTA pattern supported the decision not to pursue mechanical thrombectomy and prompted individualized blood pressure targets to preserve collateral-dependent cerebral perfusion. Because DSA was not obtained, intracranial atherosclerosis could not be formally excluded, and the diagnosis remains probable rather than definitive.

## Introduction

Moyamoya disease (MMD) is a rare, progressive, occlusive cerebrovascular disorder defined by narrowing of the terminal intracranial internal carotid arteries and proximal middle and anterior cerebral arteries, with development of a fragile network of lenticulostriate collateral vessels in response to chronic ischemia [1,2].

In adults, MMD carries a recognized risk of intracranial hemorrhage (ICH) and shows a female predominance, particularly in Western cohorts, where it is associated with autoimmune conditions including thyroid disease [3].

Although patients most often present with ischemic strokes or transient ischemic attacks (TIAs), ICH is a well-recognized and feared manifestation, attributable to the structural fragility of the moyamoya collateral vessels under elevated hemodynamic stress, and it carries significant morbidity and a heightened risk of recurrence [2]. Pathological examination of these vessels reveals pseudoaneurysm formation, internal elastic lamina disruption, and medial degeneration, the histopathological basis of ICH in this disease, and ruptured collateral-vessel aneurysms have been identified as the culprit lesion in nearly 25% of hemorrhagic cases [4,5]. The term MMD denotes the idiopathic bilateral vasculopathy, whereas moyamoya syndrome refers to an angiographically similar pattern occurring in association with a predisposing condition, such as thyroid autoimmunity, cranial irradiation, sickle-cell disease and other hemoglobinopathies, neurofibromatosis type 1, Down syndrome, or intracranial atherosclerosis, all of which should be considered when a moyamoya pattern is identified [6,7].

Digital subtraction angiography (DSA) remains the diagnostic gold standard [6] and guides revascularization planning through Suzuki staging; surgical options include direct superficial temporal artery-middle cerebral artery (STA-MCA) bypass and indirect procedures such as encephaloduroarteriosynangiosis (EDAS).

We present a 52-year-old woman with a prior TIA and hypertension who presented with bilateral M1 occlusions, a right gangliocapsular intraparenchymal hemorrhage, and a concurrent small right thalamic hemorrhage, a constellation suspicious for MMD. Management required simultaneous attention to hemorrhage control and preservation of collateral-dependent perfusion, the cardinal hemodynamic tension of acute hemorrhagic moyamoya. Evidence-based treatment guidelines for MMD remain an active area of investigation, underscoring the value of well-documented case reports [7]. Although hemorrhagic MMD is described, reports emphasizing computed tomography angiography (CTA) pattern recognition as the finding that simultaneously redirects endovascular and acute hemodynamic management remain limited.

## Case presentation

The patient was a 52-year-old Caucasian woman with a past medical history significant for hypertension, hypothyroidism, and a TIA reported approximately nine years prior. With respect to atherosclerotic risk, hypertension was her sole conventional vascular risk factor. Lipid panels were normal on this admission and had been normal historically; statin therapy had been initiated as secondary prevention after her prior TIA rather than for documented dyslipidemia. She was not obese and had no history of diabetes mellitus or tobacco use. Her hypothyroidism had been diagnosed prior to this admission and was biochemically controlled, with a normal thyroid-stimulating hormone (TSH) and free T4 on presentation. Thyroid autoantibody testing performed before this admission had been negative. Of particular clinical relevance, she had been evaluated at an outside institution several weeks before this admission for a resolved episode of TIA-like symptoms, at which time aspirin therapy was initiated. She reportedly took aspirin for approximately one week before discontinuing it prior to this presentation. The treating team considered that recent aspirin initiation, occurring in the setting of hypertensive urgency and an undiagnosed moyamoya vasculopathy, may have precipitated the ICH.

She presented to the emergency department with a two-hour history of acute-onset left-sided weakness, with a last known well time established at approximately two hours prior to arrival. Initial vital signs were notable for hypertension at 171/78 mmHg, with the remainder of the physical examination unremarkable outside of the neurological findings described below.

Admission National Institutes of Health Stroke Scale (NIHSS) was 11, indicating moderate stroke severity, with a Glasgow Coma Scale (GCS) score of 15. Examination demonstrated a left lower facial weakness with forehead sparing, consistent with a central facial palsy. There was no aphasia or visual field deficit, and she was alert and fully oriented. Strength was 5/5 throughout the right limbs, with full weakness of the left upper and lower extremities on initial assessment. Electrocardiography showed normal sinus rhythm and troponin was negative.

**Figure 1:**
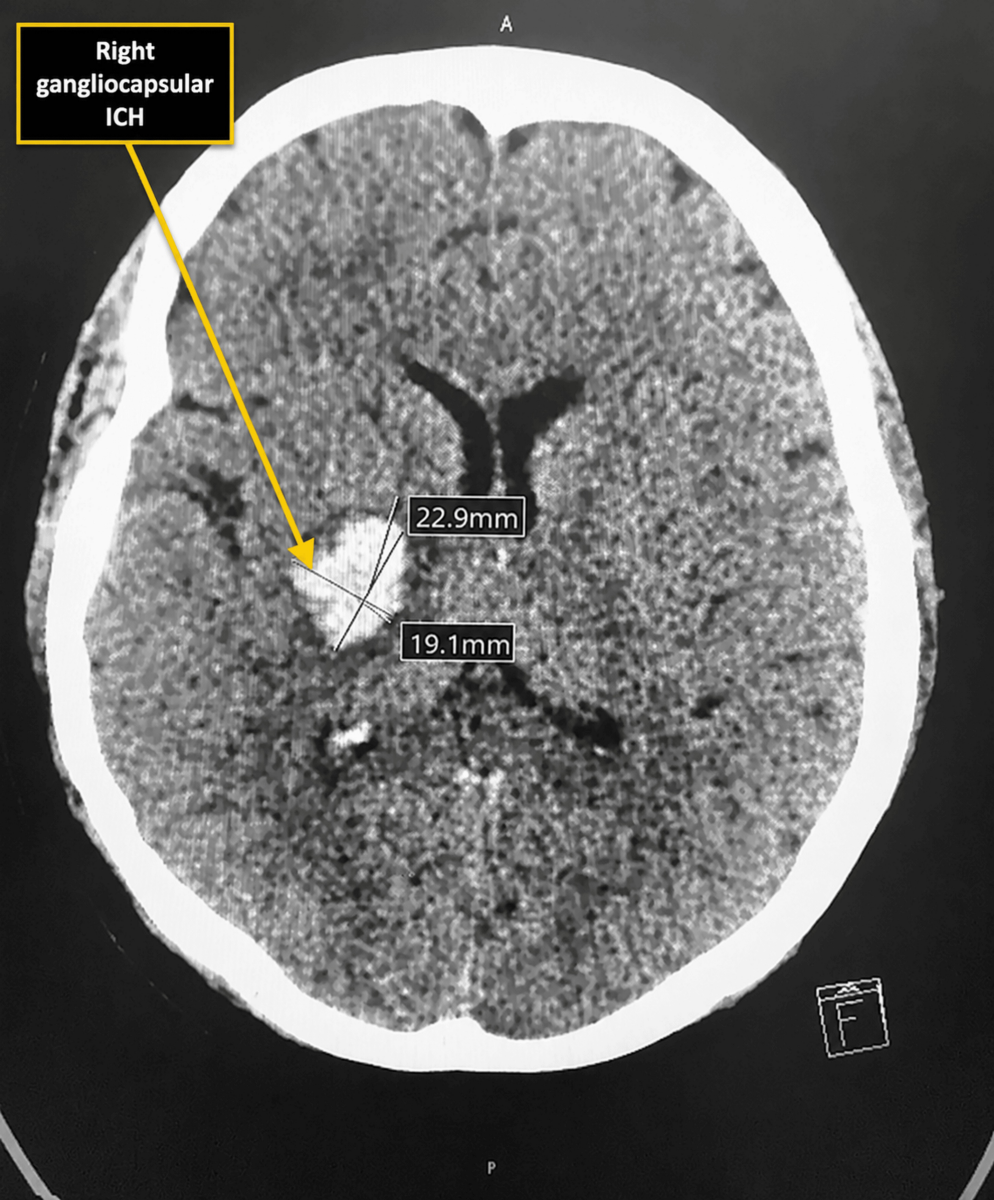
Axial non-contrast computed tomography A hyperdense intraparenchymal hemorrhage is present in the right gangliocapsular region (measuring approximately 22.9 by 19.1 mm in plane), with a smaller concurrent right-sided focus. The deep location is compatible with hemorrhage related to moyamoya collateral rupture but also overlaps with common hypertensive intracerebral hemorrhage locations; given the patient's hypertension, it is not specific and is interpreted together with the computed tomography angiography findings.

Non-contrast computed tomography (CT) of the head revealed a 2.3 × 1.9 × 2.5 cm intraparenchymal hemorrhage in the right gangliocapsular region (Figure 1) with minimal surrounding vasogenic eema and minimal localized mass effect.

Approximately 2 mm of right-to-left midline shift was noted, without herniation or hydrocephalus. A small right thalamic hemorrhage was concurrently identified. The gangliocapsular location is anatomically consistent with the patient's left hemiplegia, as the posterior limb of the internal capsule in this region carries the corticospinal and corticobulbar tracts subserving contralateral motor function.

**Figure 2:**
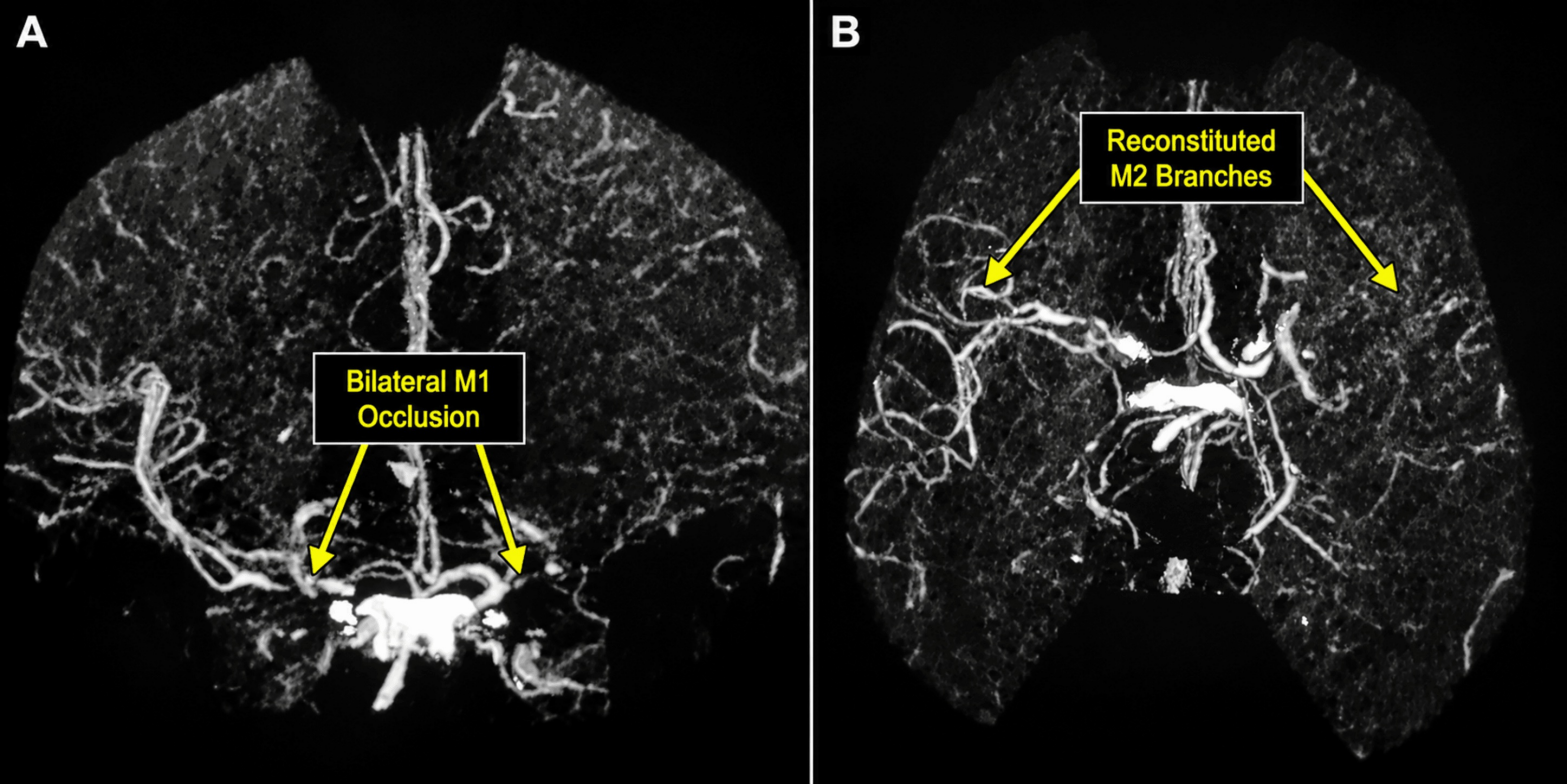
Computed tomography angiography, maximum-intensity-projection (MIP) reconstructions (A) Coronal MIP shows bilateral M1 segment occlusion, with arrows indicating the sites of the absent M1 segments at the internal carotid artery bifurcations. Attenuated anterior cerebral arteries are reconstituted via collateral pathways, and a fine network of hypertrophied lenticulostriate and basal perforating collateral vessels is present in the deep basal ganglia bilaterally, producing the characteristic puff of smoke appearance; the basilar artery and posterior circulation remain well opacified; (B) Axial MIP at a more superior level demonstrates reconstituted M2 branches (arrows), relatively preserved posterior cerebral arteries, a prominent basal collateral network, and markedly reduced cortical opacification in the bilateral middle cerebral artery territories, with faint peripheral vessels suggesting leptomeningeal collateral reconstitution. These findings indicate chronic bilateral steno-occlusive disease with extensive collateral formation rather than an acute, thrombectomy-eligible large vessel occlusion, and are suggestive of a moyamoya vasculopathy; they are not independently definitive, and DSA remains the reference standard for confirmation.

Because she presented as an acute stroke with hyperacute left hemiplegia and a last known well of approximately two hours, CTA was performed as part of the standard hyperacute stroke workup to evaluate for large vessel occlusion and to characterize the intracranial vasculature underlying the hemorrhage, particularly given her prior TIA. CTA demonstrated bilateral M1 segment occlusions with distal M2 reconstitution (Figure 2).

This pattern immediately distinguished chronic bilateral steno-occlusive disease from an acute, thrombectomy-eligible large vessel occlusion and, in the context of gangliocapsular and thalamic hemorrhage and a prior TIA, was strongly suspicious for MMD, prompting urgent neurovascular consultation.

Despite the presence of proximal large vessel occlusion, mechanical thrombectomy was not pursued. In the setting of acute neurological deficit, large vessel occlusion typically prompts emergent endovascular intervention; however, the symmetric nature of the occlusions and the presence of distal reconstitution suggested a chronic steno-occlusive process rather than an acute thromboembolic event. In MMD, endovascular thrombectomy is not indicated and may carry increased procedural risk due to fragile collateral vasculature and altered hemodynamics. Recognition of this imaging pattern therefore directly altered interventional management by preventing inappropriate endovascular intervention.

While CTA can raise diagnostic suspicion, DSA remains the gold standard for definitive moyamoya diagnosis, enabling precise characterization of steno-occlusive lesions and the moyamoya collateral network [6,7]. Formal angiography was appropriately deferred pending clinical stabilization from the acute hemorrhage, as discussed in subsequent sections.

On hospital day two, formal neurological examination documented left upper extremity strength of 0/5, with left lower extremity strength of two to three out of five proximally and 1/5 distally, indicating a dense left hemiparesis with relative proximal leg sparing. Sensation to light touch, temperature, and vibration was reduced across the left face, arm, and leg. Reflexes were diminished on the left relative to the right, with absent left finger flexors and a mute left plantar response. These findings were concordant with the right gangliocapsular and thalamic hemorrhages. Repeat non-contrast CT demonstrated a stable hematoma without interval expansion, supporting continued non-surgical management.

Neurosurgery was consulted immediately upon the patient's arrival and was notified of the imaging findings. The clinical and radiographic picture, bilateral M1 occlusions with distal reconstitution, gangliocapsular hemorrhage, thalamic hemorrhage, and a prior TIA history in a middle-aged woman with hypertension, raised immediate concern for MMD as the unifying diagnosis. Aspirin was held on admission given the active hemorrhage, and the patient was admitted to the floor with orders for serial neurological checks.

The initial hemodynamic priority on presentation was blood pressure control. Elevated admission blood pressure in the setting of acute ICH is a well-established therapeutic target: hematoma expansion occurs in up to approximately one third of ICH patients within the first 24 hours and is strongly associated with neurological deterioration and poor functional outcome [8]. The 2022 American Heart Association/American Stroke Association (AHA/ASA) Guidelines for the Management of Spontaneous Intracerebral Hemorrhage recommend that for patients with ICH and systolic blood pressure between 150 and 220 mmHg, acute lowering to a target systolic of 140 mmHg is safe and may be effective for improving functional outcome, with particular emphasis on smooth, sustained control that avoids large fluctuations in systolic pressure [9].

Intravenous nicardipine, a titratable calcium channel blocker, was selected as the agent of choice. Its use in the hyperacute ICH setting is well-supported: nicardipine served as the primary agent in the landmark Antihypertensive Treatment of Acute Cerebral Hemorrhage (ATACH-2) trial and has been demonstrated in individual participant data analyses to achieve rapid, predictable systolic blood pressure reduction with associations with reduced hematoma expansion [10,11]. Initial parameters targeted blood pressure below 140/90 mmHg with a goal of 130/80 mmHg, consistent with standard post-ICH neurology guidance at the time of admission.

On hospital day two, neurosurgery concurred with the radiographic impression of moyamoya vasculopathy, based on the characteristic CTA pattern of bilateral terminal internal carotid and proximal M1 steno-occlusion with lenticulostriate collateralization, interpreted alongside the deep hemorrhage location and prior TIA history rather than on formal diagnostic criteria or DSA. The diagnosis was therefore considered probable and imaging-based, pending DSA confirmation. This concurrence immediately reframed the blood pressure strategy. Because the collateral-dependent territories distal to the bilateral M1 occlusions have impaired autoregulation and are passively pressure-dependent, aggressive lowering toward standard ICH targets risked ischemic injury in already flow-limited MCA territories [12-14].

Recognition of the probable moyamoya pattern thus reframed blood pressure management. Standard spontaneous-ICH guidelines favor aggressive systolic reduction to limit hematoma expansion, whereas the collateral-dependent moyamoya circulation is vulnerable to hypoperfusion under those same targets. The 2021 Japanese guidelines acknowledge this tension and offer no definitive evidence-based resolution, underscoring the value of case-level documentation [15].

In response to this probable, imaging-based diagnosis, the blood pressure target was revised from the initial aggressive post-ICH parameters to a systolic range of 120 to 160 mmHg, a deliberately broader and more permissive corridor calibrated to simultaneously limit re-hemorrhage risk while preserving the minimum perfusion pressure required to sustain collateral-dependent flow. The nicardipine drip was carefully titrated within this revised framework, and the team elected not to lower blood pressure aggressively below the lower bound of this range, recognizing that hypotension in the setting of bilateral M1 occlusion would risk watershed infarction in territories already operating at marginal perfusion. By hospital day three, blood pressure was maintained stably within the target systolic range, and the nicardipine infusion was successfully discontinued. The patient was transitioned to her home antihypertensive regimen of lisinopril-hydrochlorothiazide, with continued goal-directed blood pressure management on oral agents through the remainder of the admission. Aspirin was held throughout the admission given the active ICH.

Cerebral angiography for Suzuki grading and surgical planning was the next critical diagnostic step but was appropriately deferred pending clinical recovery from the acute ICH [6,7]. The hemodynamic shifts associated with catheter-based angiography, including the effects of contrast administration, sedation, and procedural blood pressure fluctuation, carry non-trivial risk in the setting of an acute hemorrhage and bilateral flow-limiting occlusions, and the team determined that the diagnostic and surgical planning yield of angiography would be best realized after neurological stabilization. Physical therapy, occupational therapy, and speech therapy evaluations were ordered during the admission to begin early rehabilitation planning. The patient's blood pressure remained within the target systolic range through the remainder of the admission, and she was discharged to an inpatient rehabilitation facility on hospital day five following successful insurance authorization, with a plan for outpatient DSA to confirm the diagnosis and guide revascularization planning. The full hospital course, including the timing of imaging, blood pressure management, and neurosurgical decision-making, is summarized in Table 1.

**Table 1:** Timeline of presentation, imaging, blood pressure management, neurosurgical decision-making, and discharge CT, computed tomography; CTA, computed tomography angiography; ICH, intracerebral hemorrhage; LVO, large vessel occlusion; DSA, digital subtraction angiography; NIHSS, National Institutes of Health Stroke Scale; GCS, Glasgow Coma Scale.

Timepoint	Event
Presentation	Acute-onset left hemiplegia, last known well approximately two hours prior. Blood pressure 171/78 mmHg, NIHSS 11, GCS 15.
Initial imaging	Non-contrast CT: 2.3 by 1.9 by 2.5 cm right gangliocapsular ICH with small right thalamic hemorrhage, 2 mm midline shift, no herniation. CTA: bilateral M1 occlusions with distal M2 reconstitution.
Acute decision	Imaging pattern recognized as chronic bilateral steno-occlusive disease rather than thrombectomy-eligible LVO; mechanical thrombectomy withheld. Urgent neurosurgery consultation; aspirin held.
Initial BP management	Intravenous nicardipine started toward a standard post-ICH target (below 140/90 mmHg, goal 130/80 mmHg).
Hospital day 2	Repeat non-contrast CT: stable hematoma without interval expansion. Neurosurgery concurred with a probable, imaging-based diagnosis of moyamoya vasculopathy pending DSA.
Revised BP management	Target revised to a permissive systolic corridor of 120 to 160 mmHg to balance re-hemorrhage risk against collateral-dependent perfusion.
Hospital day 3	Blood pressure stable within range; nicardipine discontinued; transitioned to home lisinopril-hydrochlorothiazide. DSA deferred pending stabilization.
Hospital day 5	Neurologically stable; discharged to inpatient rehabilitation.

## Discussion

The defining challenge in this case, and the reason it merits report, was the collision of two opposed hemodynamic imperatives within a single patient, requiring a blood pressure target that neither standard guideline could supply alone. Standard AHA/ASA guidance recommends acute systolic reduction toward 140 mmHg to attenuate hematoma expansion [9]. Yet, in MMD, the collateral-dependent MCA territories have severely impaired autoregulation, so distal perfusion becomes passively dependent on systemic pressure [12]. Single-Photon Emission Computed Tomography (SPECT) perfusion studies show that reductions in mean arterial pressure of only 10 to 20% below baseline measurably decrease regional cerebral blood flow in these patients. At the same time, comparable elevations augment it [13], and intentional hypotension in advanced MMD has produced ischemic stroke [14].

The practical consequence at the bedside was that the standard 140 mmHg ceiling, applied rigidly, could have converted a stable hemorrhage into a watershed infarction in territories already operating at the margin of adequate flow. The team therefore reasoned in terms of a corridor rather than a ceiling: a lower bound near 120 mmHg to preserve collateral perfusion, and an upper bound near 160 mmHg to limit re-hemorrhage, with nicardipine titrated to hold the patient inside that window rather than driven toward a single low number. This individualized 120 to 160 mmHg range reflects a calibration for which no randomized trial provides guidance and which the 2021 Japanese guidelines acknowledge as lacking consensus [15].

The broader teaching point is that the correct blood pressure target in hemorrhagic moyamoya is not a value to be looked up but a judgment to be reasoned, anchored to the patient's collateral-dependent physiology. No perfusion imaging, autoregulatory testing, or transcranial Doppler assessment was performed to guide this target, so the selected corridor reflects individualized clinical judgment rather than an objective collateral perfusion metric, and the optimal acute blood pressure target in hemorrhagic moyamoya remains unknown. Accordingly, the management described here should be read as case-based reasoning tailored to one patient, illustrating how a target may be reasoned from collateral-dependent physiology, and not as an evidence-based recommendation or a generalizable protocol; the specific 120 to 160 mmHg corridor was an individualized choice rather than a validated threshold.

Recognizing that dilemma depended entirely on the imaging. This case represents a paradigmatic example of imaging-driven management in interventional radiology, in which CTA findings strongly suggested the underlying diagnosis and directly redirected acute management by distinguishing chronic vasculopathy from thrombectomy-eligible large vessel occlusion, thereby altering both interventional and hemodynamic decision-making. In the modern era of endovascular stroke therapy, rapid identification of large vessel occlusion typically prompts emergent mechanical thrombectomy. However, this case highlights the critical importance of recognizing imaging patterns that fall outside this paradigm, where intervention is not only unwarranted but potentially harmful.

The hemorrhagic location in this case, the right gangliocapsular region with concurrent thalamic involvement, is anatomically characteristic of MMD. In adults with MMD, hemorrhage arises chiefly from rupture of the fragile lenticulostriate collateral network that bears chronically elevated hemodynamic stress in compensation for the occluded M1 segments, accounting for its predilection for the basal ganglia and periventricular white matter [2]. Histopathological studies of these vessels confirm structurally compromised walls, with disrupted internal elastic laminae and medial degeneration, rendering them susceptible to rupture under conditions of acute hypertensive surge [4]. The long-term natural history of hemorrhagic MMD underscores the severity of this risk: rebleeding rates among nonsurgically managed patients reached 8.2% per year in the Japan Adult Moyamoya Trial, with basal ganglia hematoma representing the most common hemorrhagic subtype [16]. Among hemorrhagic presentations, rebleeding significantly worsens outcome, and blood pressure control is considered critical at both the acute and chronic stages of disease management [16,17]. Prognosis in hemorrhagic moyamoya is accordingly guarded and driven largely by the initial hemorrhage severity and by the risk of recurrence: functional outcomes vary widely, and the risk of recurrent hemorrhage is a principal determinant of long-term prognosis [16].

In the absence of angiographic confirmation, the diagnosis rests on the characteristic CTA pattern and clinical context, and CTA does not substitute for DSA in definitive diagnosis, Suzuki staging, collateral characterization, or detection of high-risk collateral aneurysms. The patient's hypothyroidism raises the possibility of an associated moyamoya syndrome; thyroid autoantibody testing obtained prior to this admission was negative, and thyroid function was normal on presentation, findings that argue against thyroid autoimmunity and therefore against a thyroid-associated moyamoya syndrome in this patient. Negative serologies do not by themselves establish idiopathic MMD, however, and definitive disease versus syndrome classification, along with Suzuki staging, awaits angiographic characterization. The bilateral moyamoya pattern also warrants consideration of alternative causes of intracranial steno-occlusive disease [6,7]. Intracranial atherosclerosis is the most important of these, as it is common, can involve the distal internal carotid and proximal middle cerebral arteries bilaterally, and may mimic a moyamoya pattern [18]; it more often produces asymmetric, focal stenoses in patients with a heavier atherosclerotic burden rather than the symmetric terminal internal carotid pattern with basal collateralization seen here, and this patient carried only a single conventional risk factor. This distinction is not absolute on CTA, however, and is reliably made only with DSA.

Inflammatory vasculitis and radiation-associated vasculopathy were not supported by the history, and there was no prior cranial irradiation. Sickle-cell and other hemoglobinopathy-associated vasculopathies were not suggested by the patient's history, and no vasculitis or hemoglobinopathy workup was obtained. The symmetric bilateral distribution, terminal internal carotid involvement with lenticulostriate collateral formation, and characteristic collateral network favor a moyamoya vasculopathy over these alternatives, and negative thyroid autoantibodies argue against the secondary form most relevant to this patient. DSA would nonetheless be required to formally exclude the remaining alternatives, particularly intracranial atherosclerosis.

This case also highlights the importance of early vascular imaging and specialist evaluation before establishing long-term antithrombotic strategies in patients with atypical or unexplained cerebrovascular events. Aspirin had been started empirically at an outside institution weeks earlier for TIA-like symptoms, without vascular imaging. In a patient with fragile moyamoya collateral vessels and uncontrolled hypertension, antiplatelet therapy is plausibly hazardous, and the treating team considered recent aspirin initiation in the setting of hypertensive urgency a possible precipitant of the hemorrhage. Although causation cannot be established from a single case, the sequence supports characterizing the intracranial vasculature before committing a patient with an unexplained cerebrovascular event to antiplatelet therapy.

The patient's hypothyroidism is notable given the recognized association between thyroid autoimmunity and MMD in Western populations [19]. In this case, however, thyroid autoantibodies were negative, making an autoimmune thyroid-associated moyamoya syndrome less likely and favoring an idiopathic vasculopathy, though this remains unconfirmed without angiography.

Although the neurosurgical team was central to the acute clinical decision-making, definitive diagnosis and disease staging remain radiological: from an interventional radiology standpoint, DSA is the definitive study for diagnosis, angiographic Suzuki staging, collateral characterization, identification of high-risk collateral aneurysms, and operative planning, and would have been the critical next step for this patient following stabilization. The evidence base for revascularization in hemorrhagic MMD is limited but meaningful: the Japan Adult Moyamoya Trial, the only randomized controlled trial of extracranial-intracranial (EC-IC) bypass in hemorrhagic MMD, showed that bilateral direct bypass reduced annual rebleeding from 8.2% to 3.2% [20], and the 2021 Japanese guidelines recommend revascularization for hemorrhagic MMD with a Grade B recommendation [15]. Given this patient's bilateral M1 disease, both hemispheres would have warranted staged consideration for revascularization, guided by DSA, perfusion imaging, and her neurological recovery, had she remained in follow-up.

The principal limitation of this report is that DSA, the current reference standard for the diagnosis of MMD, was appropriately deferred during the acute admission and planned on an outpatient basis but was never performed, because the patient was lost to follow-up after discharge. The diagnosis therefore remains probable and imaging-based, and MMD cannot be considered definitively established. A direct consequence is that intracranial atherosclerotic stenosis, which is highly prevalent, can produce bilateral steno-occlusive disease of the distal internal carotid and proximal middle cerebral arteries, and shares imaging features with moyamoya on CTA, cannot be reliably excluded in the absence of DSA [18]. In this patient, hypertension was the only conventional atherosclerotic risk factor, with normal lipid panels, no diabetes, no obesity, and no tobacco use, a profile that makes intracranial atherosclerosis less likely. A limited risk profile does not exclude it, however, and in the absence of DSA, an atherosclerotic mechanism remains a formal alternative that tempers the certainty with which a moyamoya diagnosis can be assigned on imaging alone. As a result of this loss to follow-up, functional outcome, including the modified Rankin Scale, rehabilitation trajectory, follow-up vascular imaging, the planned outpatient DSA and any revascularization decision-making, and any recurrent ischemic or hemorrhagic events could not be ascertained. DSA-based Suzuki staging, definitive diagnostic confirmation, and consideration of revascularization therefore remain undone and represent important directions that were not achievable in this case.

## Conclusions

Bilateral M1 occlusion with distal M2 reconstitution on CTA in the setting of gangliocapsular or thalamic hemorrhage should raise suspicion for MMD, regardless of ethnicity or geographic background. The same imaging pattern helps distinguish chronic vasculopathy from acute thromboembolic large vessel occlusion and, in this case, supported the decision not to pursue mechanical thrombectomy.

Recognition of moyamoya may substantially influence blood pressure management, because aggressive blood pressure lowering could threaten collateral-dependent perfusion in selected patients, and an individualized, permissive systolic range may be appropriate until DSA-guided revascularization planning is complete. This case supports heightened suspicion and individualized, physiology-anchored decision-making rather than a generalized mandate for any specific blood pressure range. When a cerebrovascular event is atypical or unexplained, vascular imaging should precede any long-term antithrombotic commitment.

DSA remains the gold standard for diagnosis, Suzuki staging, collateral aneurysm identification, and surgical planning. Deferral during the acute hemorrhagic phase is appropriate, but this case underscores that a deferred study must actually be secured, as loss to follow-up leaves the diagnosis unconfirmed. The Japan Adult Moyamoya Trial supports EC-IC bypass to reduce rebleeding and should inform long-term revascularization planning.
